# Labeling of penicillin allergy in patients admitted to a tertiary hospital in Qatar: A retrospective audit

**DOI:** 10.5339/qmj.2022.fqac.25

**Published:** 2022-04-04

**Authors:** Reem Hasan Elajez, Dana Bakdach, Rana Al-Adawi, Dina Elgaily, Ahmed Karawia, Asmaa Mohamed

**Affiliations:** ^1^Pharmacy Department, Hamad General Hospital, Hamad Medical Corporation, Doha, Qatar E-mail: relajez@hamad.qa; ^2^Pharmacy Department, Rumailah Hospital, Hamad Medical Corporation, Doha, Qatar

**Keywords:** penicillin allergy, cross-sensitivity, allergy labeling

## Abstract

Background: Penicillin (PNC) allergy is a major healthcare concern that necessitates antibiotic substitution which is associated with increased costs, worse clinical outcomes, and increased risks of antimicrobial resistance. Many patients are labeled as PNC allergic; however, this has been rarely confirmed. In this audit, we aimed to determine the characteristics of PNC allergy labeling.

Methods: A list of all the patients labeled with PNC allergy who presented to the Hamad General Hospital (HGH) for any medical reason from January to December 2021 was generated from pharmacy system. Of those, 30% were randomly selected for audit review. Electronic health records of the selected patients were retrospectively reviewed to identify the allergy labeling characteristics and whether the patients had recently received an antibiotic within the PNC class without developing any allergic reaction.

Results: Of the 464 patients identified with labelled PNC allergy, 139 patients were randomly selected and reviewed. Of the reviewed patients, 82 (59%) were women with an average ( ± SD) age of 46 ( ± 16.5) years. Forty-six patients were categorized to have a mild PNC allergy, and only 18 were categorized as severe with the remaining patients categorized as having a moderate PNC allergy. Despite documentation of severity, an accurate description of the allergic reaction event was significantly lacking with only 30/139 (21.5%) patients having clear documentation of the event description. Twenty (14.4%) patients labeled as PNC allergic received at least one antibiotic within the PNC class (e.g., piperacillin-tazobactam, ampicillin-sulbactam, or amoxicillin-clavulanic acid) safely without any documented reactions. Interestingly, of those 20 patients, 4 were categorized as being severely allergic to PNC. However, as more than 80 patients presented to the hospital for reasons not requiring antibiotics; experiences with PNC could not be assessed effectively.

Conclusion: Poor documentation of the details of allergic reactions may falsely affect future antibiotic decisions. The results of this audit highlight the need for standardizing the documentation process of medication allergy. In addition, reviewing the patient's experience with other drugs within the PNC class can guide healthcare providers during the PNC allergy evaluation.

